# Characterization of NADH fluorescence properties under one-photon excitation with respect to temperature, pH, and binding to lactate dehydrogenase

**DOI:** 10.1364/OSAC.423082

**Published:** 2021-05-10

**Authors:** Taylor M. Cannon, Joao L. Lagarto, Benjamin T. Dyer, Edwin Garcia, Douglas J. Kelly, Nicholas S. Peters, Alexander R. Lyon, Paul M. W. French, Chris Dunsby

**Affiliations:** 1 Department of Physics, Imperial College London, London, SW7 2AZ, UK; 2 National Heart and Lung Institute, Imperial College London, Du Cane Road, London, W12 0NN, UK; 3 Centre for Pathology, Imperial College London, London, W12 0NN, UK; 4 These authors contributed equally to this work and are listed in alphabetical order

## Abstract

Reduced nicotinamide adenine dinucleotide (NADH) is the principal electron donor in glycolysis and oxidative metabolism and is thus recognized as a key biomarker for probing metabolic state. While the fluorescence characteristics of NADH have been investigated extensively, there are discrepancies in the published data due to diverse experimental conditions, instrumentation and microenvironmental parameters that can affect NADH fluorescence. Using a cuvette-based time-resolved spectrofluorimeter employing one-photon excitation at 375 nm, we characterized the fluorescence intensity, lifetime, spectral response, anisotropy and time-resolved anisotropy of NADH in aqueous solution under varying microenvironmental conditions, namely temperature, pH, and binding to lactate dehydrogenase (LDH). Our results demonstrate how temperature, pH, and binding partners each impact the fluorescence signature of NADH and highlight the complexity of the fluorescence data when different parameters produce competing effects. We hope that the data presented in this study will provide a reference for potential sources of variation in experiments measuring NADH fluorescence.

## 1. Introduction

Reduced nicotinamide adenine dinucleotide (NADH) is an endogenous fluorophore with excitation and emission wavelength bands in the ranges 320-380 nm and 420-480 nm, respectively. Its fluorescence has been measured in aqueous solution [1], cells [2–4], tissues [5,6], and *in vivo* live organisms [7,8]. For a complete background, see the review by Blacker and Duchen [9]. As it is a metabolic coenzyme, NADH fluorescence has been investigated by researchers seeking to detect and monitor metabolic changes, including in longitudinal studies, without the introduction of exogenous labels or dyes.

Fluorescence can be parametrized by its intensity, spectral, temporal, and polarisation properties. In terms of fluorescence intensity, the most significant change in NADH fluorescence signal is caused when NADH is oxidized to NAD^+^ during metabolic processes. NAD^+^ presents a single excitation maximum at around 260 nm and is not efficiently excited at wavelengths longer than 320 nm (see Fig. 1(B) in Ref. [6]); NADH presents two excitation maxima at 260 nm and 340 nm. Therefore, changes in NADH fluorescence intensity (excited at e.g. 340 nm) can be used to infer changes in the redox state. However, absolute measurements of fluorescence intensity depend on the specific experimental geometry employed for fluorescence excitation and detection and the optical properties of the sample, and therefore require careful calibration, making it challenging to compare results between different experimental set-ups and laboratories. Ratiometric intensity measurements comparing intensities measured under two different excitation and/or emission wavelengths can be used to eliminate many of the unknown quantities, and the approach of calculating the ratio of NADH fluorescence to the fluorescence from flavins, i.e. calculation of the optical redox ratio, was first pioneered by Chance *et al.* [10]. Measurements of the redox ratio can, however, be confounded by further complexities of cellular autofluorescence. These factors include fluorescence arising from reduced nicotinamide adenine dinucleotide phosphate (NADPH), which is spectrally identical to NADH in its unbound form [11,12], but biochemically and functionally distinct. Cellular and tissue autofluorescence, where NADH and NADPH cannot be distinguished, is therefore often referred to as NAD(P)H emission [13]. Another potential confounding factor is that the fluorescence quantum yield of NADH can change when it binds to its cofactors. For example, it increases ∼two-fold when bound to malate dehydrogenase (MDH) [14], ∼3.3-fold when bound to lactate dehydrogenase (LDH) [1], and decreases by a factor of ∼1.7 when bound to glyceraldehyde 3-phosphate dehydrogenase (GAPDH) [1]. NADPH fluorescence also increases when bound to MDH [15]. In terms of the NADH emission spectrum, the fluorescence peak has been shown to remain approximately unchanged when bound to MDH [14] or GAPDH [1], and to blue-shift by ∼25-30 nm when bound to LDH [1,16] or octopine dehydrogenase (ODH) [17] (see column 4 of Table 1). Table 1 summarizes measured fluorescence intensity properties of free and enzyme-bound NADH over different studies. For a more in-depth analysis, see also references 5-15 within the paper by Galeotti *et al.* [13].

**Table 1.** Summary of some measurements of free and enzyme-bound NADH fluorescence intensity. N.B. a complete review of all such measurements is beyond the scope of this paper.

1^st^ author and year	Fluoro-phore	λ_abs-max_ (nm)	λ_ex-max_ (nm)	λ_em-max_ (nm)	Temperature (^o^C)	Solvent	pH	Reference
Velick 1958	NADH		350	465	25	Tris	7.1	[1]
Scott 1970	NADH		340	470	25	^*a*^		[16]
Lackowicz 1992	NADH			450-470	24	Mops buffer	7	[14]
Brochon 1977	NADH + ODH	340	336	466	10	PPB	7.1	[17]
Kierdaszuk 1996	NADH			460	20	Tris	7.5	[18]
Fjeld 2003	NADH			455	25	Tris	8	[19]
Pu 2010	NADH	345		462		Aq. soln		[20]
Torikata 1979	NADH + MDH	345			25	Tris	8	[21]
Lackowicz 1992	NADH + MDH			470	24	Mops buffer	7	[14]
Velick 1958	NADH + LDH		350	440	25	Tris	7.1	[1]
Scott 1970	NADH + LDH		335	440		PBS	7	[16]
Torikata 1979	NADH + LDH	345			25	Tris	8	[21]

^*a*^ unclear butter (Tris, pH = 8 or H_2_O, pH = 7); PBS—phosphate buffered saline; PPB—potassium phosphate buffer

The fluorescence decay properties of NADH have also been extensively studied. When free in aqueous solution, NADH exists in folded and extended forms [16], and has been reported to have a double exponential decay profile with decay components (pre-exponential fractions) of: 250 ps (0.82) and 690 ps (0.18) [11]; 280 ps (0.68), 620 ps (0.32) and 1800 ps (<0.1%) [22]; and 350 ps (0.77) and 760 ps (0.23) [23]. The long and short decay components have been attributed to the folded and extended populations respectively [11,22], but later reports attribute both components to the dihydronicotinamide chromophore [18,24,25].

The fluorescence decay profiles of binary complexes of protein-bound NADH have also been found to exhibit complex exponential decay profiles. When bound to LDH, the following decay components (pre-exponential fractions) have been reported: ∼1.7 ns (0.54) and ∼4 ns (0.46) for [LAD binding site]/[NADH] = 3.3 [26]; and 0.23 ns (0.44), 0.51 ns (0.52) and 1.59 ns (0.04) for [NADH]:[LDH] = 32:1 [27]. In the case of octopine dehydrogenase, decay components 1.2 ns (0.46) and 3.1 ns (0.54) [17] were reported. Values for mitochondrial MDH (mMDH) have been measured using two-photon excitation as: 0.6 ns (0.72) and 1.33 ns (0.28) [23]; and 0.36 ns (0.42), 0.65 ns (0.51) and 1.57 ns (0.06) for [NADH]:[mMDH] = 16:1 [27]. The fluorescence lifetimes of tertiary complexes of LDH-NADH-isobutyramide have also been reported to be double exponential: 2.9 ns (0.29) and 6.9 ns (0.71) [26]; and 0.68 ns (0.12) and 4.1 ns (0.88) [18].

Changes in cellular NAD(P)H fluorescence lifetime associated with a number of environmental factors have been reported, including confluence and serum starvation in 2D culture [28], hypoxia [23], cyanide [28,29], glucose [30], rotenone [30], staurosporine-induced apoptosis [31], stem cell differentiation [32–34], and ouabain [35]. Changes in NAD(P)H fluorescence lifetime have also been associated with a number of disease states, including cancer *in vitro* [36,37] and *in vivo* [8,38]. A common method for analysing NAD(P)H fluorescence decays obtained from isolated mitochondria, cells, and tissues is to fit the decay data to a double exponential model and then associate the short decay component with free NAD(P)H and the long decay component with protein-bound NAD(P)H. Under this assumption, the normalized pre-exponential factors provide information on the relative concentrations of free and protein-bound NAD(P)H. However, in reality, the underlying decay will contain two decay components from free NAD(P)H and (at least) two additional decay components from each protein-bound form of NADH or NADPH that is present. Sometimes, the NADPH fluorescence signal is assumed to be small relative to that of NADH [23], and often changes in the NAD(P)H fluorescence lifetime are attributed to a change in the relative contributions of oxidative phosphorylation and glycolysis to cellular ATP production. However, recent work has shown that this interpretation is too simplistic [9,39,40] and that the relative concentration and fluorescence lifetime of protein-bound NADPH must be considered as well.

Despite the high volume of work published in this field, it remains difficult to compare the fluorescent properties of NADH across different studies. The challenge arises from variations in individual protocols, such as the temperature at which the experiments were conducted or the pH of the buffer used to dissolve the NADH, which affect NADH fluorescence behaviour (Table 2).

**Table 2.** Summary of solution-phase measurements of the fluorescence lifetime of NADH.

1^st^ author and year	τ_phase_ (ps)	τ_1_ (ps)	τ_2_ (ps)	τ_3_ (ps)	Temper-ature (^o^C)	Solvent	pH	Ref.
Scott 1970	400				25	^*a*^		[16]
Visser 1981		250	690		20	Sodium phosphate buffer	7	[11]
Krishnamoorthy 1987		210	530		25	PBS	7	[24]
Lakowicz 1992	370-380				24	Mops buffer	7	[14]
Schneckenberger 1992		500-600	1400-2000			Aq. Soln.	7.4	[3]
Couprie 1994		280	620	1800	20	Aq. Soln.	8	[22]
Wakita 1995		230-240	500-510	1100-1400	23	^*b*^	7.4	[41]
Kierdaszuk 1996		270	590		20	Tris	7.5	[18]
Evans 2005		280	520		RT	PBS	7.4	[30]
		140	480		RT	H_2_O	7	[30]
Vishwasrao 2005		350	760			Aq. Soln.		[23]
Zelent 2007		160	480	2400	20	H_2_O	7	[42]
Yu 2009		360	750			PBS	7.4	[27]
Blacker 2019		382	720		21	Aq. Soln.	7.2	[25]

*RT—room temperature

^*a*^ unclear buffer (Tris, pH = 8 or H_2_O, pH = 7)

^*b*^ 0.25 M mannitol, 10 mM Tris-HCl, and 1 mM EGTA

In this paper, a cuvette-based time-correlated single photoncounting (TCSPC) system developed previously in our laboratory [43] was used to carry out solution-phase fluorescence lifetime measurements of NADH under a variety of microenvironmental conditions. The raw experimental data from these measurements are available in at [44]. In order to study the experimental error, two separate datasets (Dataset 1 and Dataset 2) were acquired independently by different researchers (TC and JL/BD) using different batches of reagents and different instrumentation configurations with similar functionality. We hope that these data will help provide a better understanding of how each of the individual parameters studied impacts the fluorescent properties of NADH and provide estimates of the measurement uncertainties. Finally, we note that this manuscript provides data from NADH measurements alone. A similar study on NADPH is out of the scope of this work and will be the subject of future investigations.

## 2. Materials and methods

### 2.1 Preparation of NADH solutions

Solutions of β-NADH (N4505 (Dataset 1) and N8129 (Dataset 2), Sigma Aldrich) in phosphate buffered saline (PBS, Gibco) were freshly prepared on each day of experiments. To minimise photobleaching via natural room light, stock solutions were shielded in metal foil. All temperature and pH experiments were carried out with either 50 µM (Dataset 1) or 100 µM (Dataset 2) NADH solutions. The concentrations used were chosen by the individual experiments (JL/BD and TC) to provide a reasonable fluorescence signal while achieving low absorption of the excitation light as it passes through the cuvette.

For experiments where the temperature was varied, at each measured point the sample in the cuvette was replaced by a fresh aliquot of the stock solution to prevent photobleaching artefacts. The temperature of each new aliquot was allowed to stabilise for 15 minutes before making the fluorescence measurement. For all measurements described below we used quartz cuvettes (Helma QS) with internal dimensions of 10 × 3 mm.

Two protocols were used to adjust the pH of NADH solutions for pH experiments. In one protocol, standard pH buffer solutions were prepared from Chemvelopes (Hydrion) in distilled water, to which concentrated NADH solutions were added. This protocol was used for measurements realized at 25°C in Dataset 1 and for one set of measurements in Dataset 2 at both 25°C and 37°C. Under the second protocol, the pH of NADH solutions was adjusted directly using 0.1 M hydrochloric acid (HCl, Sigma Aldrich) and 0.1 M sodium hydroxide (NaOH, Sigma Aldrich) stock solutions. This protocol was used for the 37°C measurements of Dataset 1 and for two sets of measurements in Dataset 2 at both temperatures. The pH of each NADH solution was verified with a pH meter (Thermo Fisher Scientific) after preparation. All pH measurements were made at both 25°C and 37°C.

Lactate dehydrogenase (LDH, Sigma-Aldrich) was used to simulate the protein-bound state of NADH in a cellular environment. LDH suspensions were purified using a centrifugal protein purification system and a protocol supplied by the system’s manufacturer (Amicon Pro, Sigma Aldrich) prior to dissolution in PBS. For full information regarding LDH preparation, see page 8 of the manufacturer’s protocol for desalting/buffer exchange (Amicon Pro Pocket Brochure, [45]). The concentration of the protein was varied against that of NADH at ratios ranging from 0 to 4, where [LDH]:[NADH] = 4 should saturate the binding sites [46]. Stock solution concentrations were determined using a spectrophotometer (UV Probe, Shimadzu) and an extinction coefficient value of 16.2 × 10^4^ M^-1^cm^-1^ for LDH at 280 nm [47]. The measured concentration value was cross-checked against theoretical concentrations estimated using the molar masses of the stock LDH provided by the manufacturer and the known volume of solvent (PBS) in which the protein was dissolved. The concentration was validated by additional absorption measurements over the course of a serial dilution. For all LDH experiments, the NADH concentration was held constant at 25 µM for Dataset 1 and 12.5 µM for Dataset 2. Lower concentrations of NADH were used for the LDH-NADH experiments compared to those used in the temperature and pH experiments to reduce the amount of LDH required. Unless otherwise indicated, these experiments were carried out at 37°C. Solutions of protein were also characterized independently to provide a background signal for subtraction during analysis. Additional fluorescence lifetime measurements were carried out to investigate the temperature (at 25 and 37°C) and pH dependence of LDH-bound NADH. For these measurements, pH was adjusted between 5.5 and 9.0 using the NaOH titration method described above.

### 2.2 Time-resolved cuvette-based spectrofluorimeter setup

All fluorescence lifetime measurements were made using a previously developed custom-built, cuvette-based time-resolved fluorescence spectroscopy system [43]. The modified set-up used for these experiments is shown in Figure S1 in Supplement 1. A 375 nm pulsed diode laser (LDH-P-C-375B, PicoQuant) operated at 40 MHz was used to provide fluorescence excitation. The excitation beam was passed through a neutral density rotatable filter wheel and a polariser to control the beam intensity and polarisation state, respectively, incident on the cuvette containing the sample. Additionally, 1% of the excitation beam was reflected onto a photodiode and spectrometer (Ocean Optics) for time-averaged laser power and spectral stability monitoring. Fluorescence emission from the sample in the cuvette was passed through a rotatable emission polariser, a 450/50 nm emission filter, and a motorized monochromator (CVI CM110 with 1200 lp.mm^-1^ grating) set to 460 nm. The monochromator entrance and exit slits were 1.2 mm wide. The emission polariser was rotated through vertical, horizontal, and magic angle (54.7^o^) orientations to enable fluorescence anisotropy analysis. For Dataset 1, data were collected with a cooled photomultiplier tube (PMT, PMC-100, Becker & Hickl). For Dataset 2, a hybrid photomultiplier tube with a faster response time was used (HPM-100-06, Becker & Hickl). In both cases, the PMT was connected to TCSPC electronics (SPC730, Becker & Hickl). Fluorescence decays were accumulated over integration times between two and ten seconds. The integration time was fixed at 2 s for all measurements in Dataset 2. In Dataset 1, the integration time was fixed at 5 s for all temperature and pH experiments, and 10 s for all LDH experiments. To compare the detector performance, samples of Stilbene 3 in PBS were measured with both experimental systems and the mean lifetime results agreed to within 7 ps, which is smaller than the variation observed in NADH τ_m_ over the conditions reported.

### 2.3 Data analysis

A custom-written program (LabVIEW, National Instruments) was used for data acquisition. The data were then exported to FLIM*fit* (https://flimfit.org), an open-source time-resolved analysis software package previously developed in our laboratory [48]. Decay data from solutions of NADH were fitted using a nonlinear least squares fitting algorithm to a double exponential decay model, $I(t)=I_{0}\left(\alpha_{1}e^{-\frac{t}{\tau_{1}}}+\alpha_{2}e^{-\frac{t}{\tau_{2}}}\right)+C$ , where *I(t)* is signal intensity at time *t*, *I_0_* is the intensity at *t *= 0, $\alpha_{1}$ and $\alpha_{2}$ are the fractional pre-exponential factors (i.e. $\alpha_{1}+\alpha_{2}=1$ ) of the short ( $\tau_{1}$ ) and long ( $\tau_{2}$ ) lifetimes, respectively, and *C* accounts for constant background signal. The fit model also included incomplete decay estimation. A detailed description of the fitting algorithm is provided in Ref. [48]. The decay analysis was performed over a temporal range of 11-15 ns. The total fluorescence signal was calculated as the sum ofphotons in all time bins. The mean fluorescence lifetime, $\tau_{m}$ , was calculated as the mean of its intensity-weighted long and short components, $\left(\alpha_{1}\tau_{1}^{2}+\alpha_{2}\tau_{2}^{2}\right)$ / $\left(\alpha_{1}\tau_{1}+\alpha_{2}\tau_{2}\right)$ . Fluorescence intensity measurements were normalized to the maximum value of each experiment for comparison across multiple experiments and datasets. For protein-bound NADH experiments, the time-resolved background signal measured from a cuvette of pure protein solution was subtracted to remove a small contribution of scattered signal. The instrument response function (IRF) was measured daily using a scattering sample (2.2 µM LUDOX HS-40, Sigma Aldrich), with the emission filter removed and with the emission monochromator set to 375 nm. The fluorescence decay model was convolved with the IRF during fitting. The temporal shift of the IRF relative to the measured decay, *t_0_*, was determined as a fitted parameter when fitting the decay of free NADH and then subsequently fixed for the analysis of all other decays from the same day. An exemplar fit to a decay measured from free NADH at 25°C and at pH 7.4 is shown in Figure S2 in Supplement 1.

All reported intensity and lifetime values are from data obtained with the linear emission polarizer at the “magic angle” orientation [49] in order to remove any fluorescence anisotropy effects from the measured fluorescence decay. Steady-state fluorescence anisotropy was calculated as *r* = (*I*_ll_ - *GI*_⊥_)/(*I*_ll_ + 2*GI*_⊥_), where *I*_ll_ is the steady-state vertically-polarized emission under vertically-polarized excitation, *I*_⊥_ is steady-state horizontally-polarized emission under vertically-polarized excitation, and *G* is the G-factor, a constant parameter quantifying the system’s polarisation bias. The G-factor was calculated as *G *= *I*’_ll_/*I*’_⊥_, where *I*’_II_ and *I*’_⊥_ are the steady-state intensity of vertically and horizontally polarized emitted light respectively for a fluorescent sample excited by a horizontally polarized light. The G-factor of the system was measured on each day of experiments and verified to be constant over time. For time-resolved anisotropy fitting, FLIMfit [48] was used to determine the fluorescence decay parameters from magic angle decay fits and these were then fixed for subsequent global fitting of the parallel and perpendicular decays. A single exponential anisotropy decay model was used to analyse the fully LDH-bound NADH data, where the rotational correlation time is the time taken for the fluorescence anisotropy to decay to 1/*e* of its initial value. Exemplar magic angle, vertical, horizontal, and calculated time-resolved anisotropy decays are shown in Figure S3 to illustrate the polarisation-resolved fitting procedure used in the FLIM*fit* software.

## 3. Results

### 3.1 Temperature dependence of NADH fluorescence

**Fig. 1.**
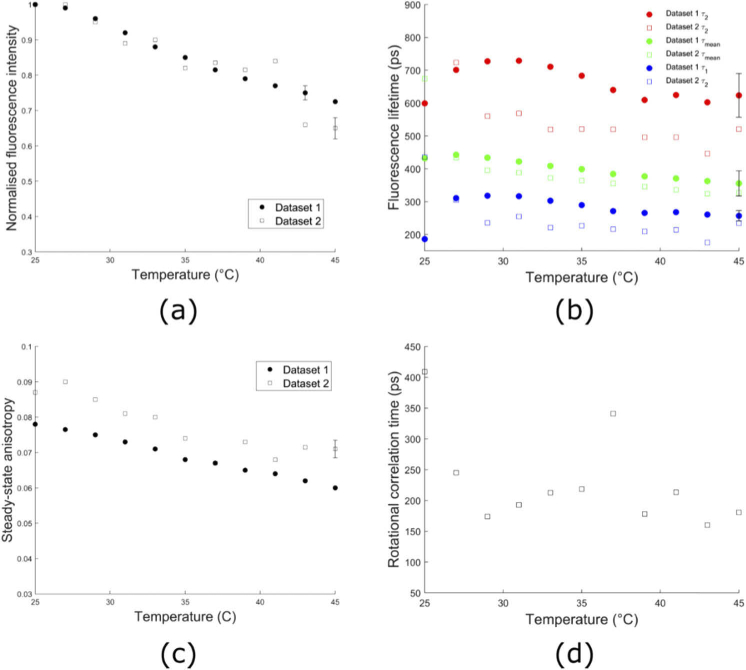
Temperature dependence of fluorescence intensity, lifetime, steady-state anisotropy and rotational correlation time for free NADH. a) The fluorescence intensity, b) long ( $\tau_{2}$ ), short ( $\tau_{1}$ ), and mean ( $\tau_{m}$ ) fluorescence lifetimes, (c) steady-state anisotropy, and (d) rotational correlation time are shown as a function of temperature. Data points in (a) and (b) show the mean of three measurements for Dataset 1 and one measurement for Dataset 2. Only one measurement of steady-state anisotropy was performed in Datasets 1 and 2 and so no error bars are shown in panel (c). Polarisation-resolved data was not obtained for Dataset 1 and so panel (d) only shows the results from Dataset 2. Where shown, error bars indicate the average over all temperatures of the standard deviation at each temperature for a specific dataset. The average error bar is shown on only one data point per Dataset to avoid the figure becoming excessively crowded.

The dependence of the fluorescence decay profile of free NADH on temperature was investigated over the range 25°C to 45°C. Previous studies have reported a decrease in the quantum yield of NADH with increasing temperature [10]. As expected, the normalized emission signal from the sample decreased as the temperature increased (Fig. 1(a)). The rate of change in normalized intensity was found to be -1.7%/^o^C in Dataset 1 and -2.8%/^o^C in Dataset 2 over the temperature range 25-45°C (determined by calculating the average slope over the data range). The overall decrease in intensity cannot be attributed to photobleaching, as a fresh aliquot of NADH was used for each measurement.

The fluorescence lifetime of NADH is also known to depend on temperature [11,16,22]. We observed a linear change of -4.6 ps/^o^C in the mean fluorescence lifetime ( $\tau_{m}$ ) of Dataset 1 and -11.2 ps/^o^C in Dataset 2 over the temperature range 25-45°C (Fig. 1(b)). In particular, the values of NADH mean lifetime at 25°C and 37°C for Dataset 1 were found to be 432 ± 38 ps and 383 ± 22 ps, respectively. Although the long ( $\tau_{2}$ ) and short ( $\tau_{1}$ ) lifetimes were more variable between datasets and the changes were less uniform over the temperature range studied compared to the mean lifetime, the overall trends remained similar.

Changes in the steady-state anisotropy of free NADH were also examined in these experiments (Fig. 1(c)). The steady-state anisotropy *r* changed at a rate of -9.1 × 10^−4^/^o^C for Dataset 1, and -10.6 × 10^−4^/^o^C for Dataset 2, over the temperature range studied. The values measured for Dataset 2 were greater than corresponding values in Dataset 1 by an average of 0.008. The rotational correlation time of free NADH as a function of temperature is shown in Fig. 1(d).

### 3.2 pH dependence of NADH fluorescence

**Fig. 2.**
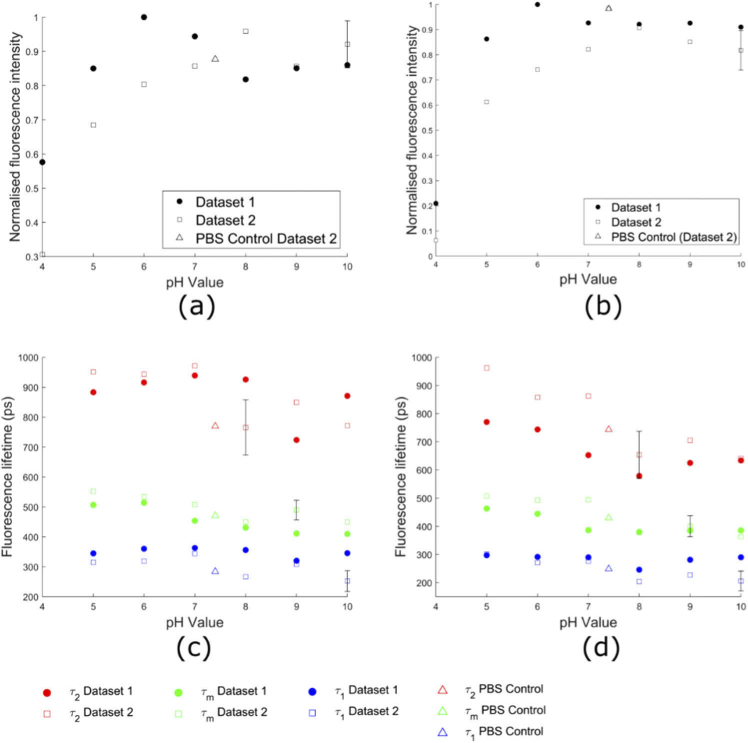
pH dependence of NADH fluorescence intensity and lifetime values at 25°C and 37°C. The fluorescence intensity as a function of pH is shown at (1) 25°C and (b) 37°C. The long ( $\tau_{2}$ ), short ( $\tau_{1}$ ), and mean ( $\tau_{m}$ ) fluorescence lifetimes of free NADH are shown at (c) 25°C and (d) 37°C. Triangles denote control samples in PBS at pH 7.4. Dataset 1 consists of one measurement and Dataset 2 consists of three measurements. For Dataset 2, we calculated the standard deviation over the three measurements at each point. To avoid overcrowding the figure, we present the average standard deviation over all pH values on just one data point.

The fluorescence intensity and lifetime of free NADH were also investigated as a function of pH, another microenvironmental parameter known to affect its fluorescence properties [50]. These experiments, over which the pH was varied from 4 to 10, were conducted at both 25°C and 37°C. At both temperatures, the normalized fluorescence intensities in both datasets decreased below pH 6 and were reasonably constant above this pH (Fig. 2(a, b)).

At pH 5 and higher, the fluorescence decay parameters of NADH are reasonably constant at both 25°C and 37°C. Below pH 5, the mean fluorescence lifetime increased for one measurement at 25°C and in all measurements at 37°C (Fig. S4). The lifetime data for pH 4 solutions were excluded from Fig. 2(c, d) as changes in lifetime with exposure to an acidic environment were found to be highly time dependent (Fig. S5(b)). This effect was only observed at pH 4. Over the pH range of 5 to 10, there is a trend for $\tau_{2}$ and $\tau_{m}$ to decrease with increasing pH, which is seen in both datasets at both temperature (Fig. 2(c, d)). The mean lifetimes at pH 7.4 for 25°C and 37°C were 471 ± 83 ps and 415 ± 79 ps, respectively, which are in good agreement with lifetimes measured at the same pH in the temperature experiments (Fig. 1(b), Table S1 in Supplement 1).

### 3.3 Lactate dehydrogenase (LDH)

**Fig. 3.**
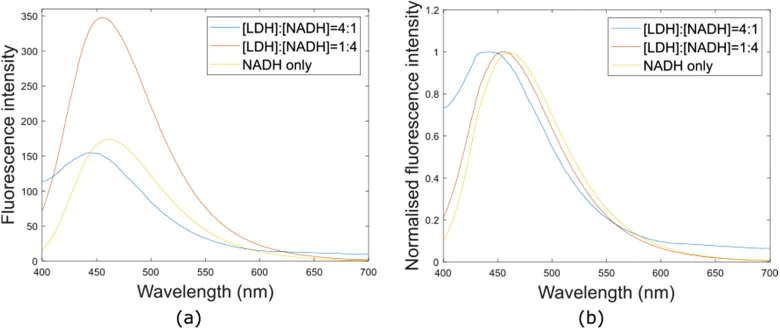
Attenuation coefficient and fluorescence emission spectra of free and LDH-bound NADH at room temperature. The emission spectra of free and LDH-bound NADH are shown as raw (a) and intensity-normalized (b) curves, with NADH concentration held constant for each sample at 12.5 μ M.

As previously reported [14], the emission spectrum of NADH is blue-shifted upon binding to LDH (Fig. 3(a)). At a lower ratio of LDH concentration to that of NADH (1:4), the intensity approximately doubled and the peak emission wavelength decreased by 7 nm. At the saturation ratio (4:1), the peak wavelength decreased by 19 nm, but signal intensity decreased back to level seen in the free NADH sample. We attribute this to an increase in optical scattering of the sample. The peak emission wavelengths are summarized in Table S2 in Supplement 1.

**Fig. 4.**
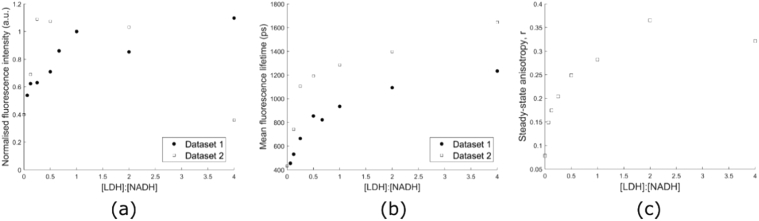
Dependence of LDH-bound NADH fluorescence intensity, lifetime, and anisotropy on protein concentration. The fluorescence intensity (a), mean lifetime ( $\tau_{m}$ ) (b), and steady-state anisotropy (c) of LDH-bound NADH are shown as a function of protein concentration. Both Datasets consist of one measurement. In (c), no LDH concentration dependent anisotropy data was taken for Dataset 1. Fluorescence intensity data are normalized to [LDH]:[NADH] = 1 intensity.

The fluorescence intensity, lifetime, and steady-state anisotropy of LDH-bound NADH at varying LDH concentrations are shown in Fig. 4. The intensity of LDH-bound NADH was seen to increase with increasing protein concentration until the concentrations of each reagent were equal (Fig. 4(a)). In both datasets, the signal intensity decreased at a ratio of [LDH]:[NADH] = 2:1. However, whereas the fluorescence intensity of the saturated LDH-NADH complex increased to a maximal value in Dataset 1, it decreased in Dataset 2 to a lower value. Again, we attribute these differences at higher protein concentrations to optical scattering of the sample.

The mean fluorescence lifetime of LDH-bound NADH increased with increasing protein concentration. Across both datasets, the average $\tau_{m}$ of fully LDH-bound NADH was found to be 1342 ± 237 ps (Fig. 4(b), average for [LDH]:[NADH] over the range 2-4). The average *τ*_1_, *τ*_2_, *α*_1_, and *α*_2_ obtained for fully LDH-bound NADH were 804 ± 306 ps, 2503 ± 831 ps, 0.85 ± 0.07 and 0.15 ± 0.07, respectively. The mean lifetime value for Dataset 2 in these experiments for free NADH ([LDH]:[NADH]=0) was 390 ± 61 ps, which is in agreement with the 37°C NADH measurements at pH 7.4 in all previously detailed results (See Table S1 in Supplement 1).

We compared the fraction of protein-bound NADH returned from fitting a quadruple exponential decay. The fluorescence lifetimes of the first two decay components and the ratio of the amplitudes of the first two decay components were fixed to those of free NADH. The last two decay components and the ratio of their amplitudes was fixed to that of fully LDH-bound NADH. The only fit parameter was the relative contribution of the free and protein-bound components and this was compared to the fraction of protein-bound NADH expected from a model of the dissociation constant for LDH and NADH [47]. In Figure S6, the theory and experimental data follow a similar trend as expected.

The steady-state anisotropy of LDH-bound NADH was also determined for each sample in Dataset 2. No polarisation resolved measurements were made for Dataset 1. Greater LDH concentrations produced greater *r* values (Fig. 4(c)). The steady-state anisotropy of fully LDH-bound NADH (for [LDH]:[NADH] = 4) was found to be 0.32. The rotational correlation time of fully LDH-bound NADH was found to be 15,900 ± 2,100 ps when fitted using a single exponential anisotropy decay. This value is greater than that of free NADH at 37°C by a factor of ∼47.

**Fig. 5.**
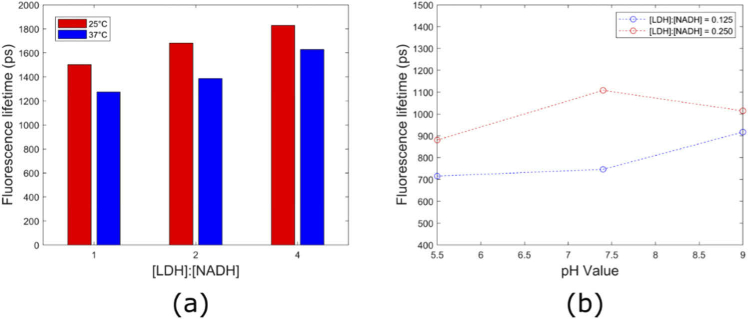
Temperature and pH dependence of LDH-bound NADH. (a) Mean fluorescence lifetime of LDH-bound NADH measured at 25°C and 37°C. (b) $\tau_{m}$ of LDH-bound NADH as a function of pH at 37°C. Each measurement was performed once.

Finally, the mean fluorescence lifetime of LDH-bound NADH was measured with respect to temperature and pH levels. Here, pH was adjusted using the NaOH titration method. The $\tau_{m}$ of LDH-bound NADH was higher at 25°C compared to 37°C, with an average increase of 241 ps over the protein-to-coenzyme ratios of 1, 2, and 4 (Fig. 5(a)). In general, the $\tau_{m}$ of fully LDH-bound NADH increased with increasing pH (Fig. 5(b)), which is the opposite of what was seen for free NADH $\tau_{m}$ (Fig. 2(d)).

## 4. Discussion

In this study, we investigated the impact of different microenvironmental conditions on the autofluorescence of NADH, including temperature, pH and binding partner (LDH), with the aim of providing a better understanding of how each of these parameters can impact NADH autofluorescence properties. To improve robustness, two sets of data were measured independently and their results were compared. The differences between Datasets 1 and 2 are the concentration, the TCSPC detected used (PMC-100 and HPM-100-06, respectively) and the experimenter (JL/BD and TC, respectively). For the temperature and pH experiments, the concentrations of NADH in solution were 50 µM and 100 µM respectively. For the LDH experiments, the concentrations of NADH in solution were 25 µM and 12.5 µM respectively.

With respect to the fluorescence intensity decay, free NADH at 25°C exhibits a complex profile with two components of 266 ± 25 ps and 781 ± 16 ps (mean ± std of independent experiments as listed in Table S1 in Supplement 1), yielding an average lifetime of 482 ± 16 ps, in agreement with previous work [11,22,23]. In our temperature-resolved experiments (see Fig. 1), our data also agreed with previously reported decreases in free NADH fluorescence intensity [10] and lifetime [11,16,22] with increasing temperature. In general, an increase in temperature is accompanied by decreases in fluorescence lifetime and quantum yield due to increased efficiency of non-radiative processes related to thermal effects such as collisions with solvent molecules, intramolecular vibrations, and rotations [51]. At 37°C the average lifetime was 397 ± 17 ps (mean ± std of independent experiments as listed in Table S1 in Supplement 1), with short and long decay components of 239 ± 27 ps and 646 ± 94 ps. The observed decrease in rotational correlation time with increasing temperature also agreed with previous reports [22]. In our experiments, there were slight discrepancies in lifetime and steady-state anisotropy values obtained between the two independently-acquired Datasets. In part, these differences could be attributed to the use of a different photomultiplier tube for these experiments. The hybrid PMT used in Dataset 2 measurements (IRF FWHM < 35 ps) has a faster response time than the cooled PMT used in Dataset 1 (IRF FWHM < 200 ps), thereby producing a narrower IRF and improved timing resolution that could have impacted the ability to measure short lifetime components. In general, we observed shorter lifetime values in Dataset 2 than those in Dataset 1. We also observed slightly higher steady-state anisotropy values in Dataset 2 compared to Dataset 1. As described above, the two datasets differ in the TCSPC detector used and in the concentration of NADH (50 µM in Dataset 1 compared to 100 µM in Dataset 2). However, these differences are not expected to affect the measurements. Further investigation is required to understand the cause of this small variation.

Our pH experiments (see Fig. 2) were also in agreement with previous studies examining the effects of acidic and basic environments on the optical properties of free NADH [50]. Our results suggest a decrease in lifetime with increasing pH at both 25 and 37°C. Interestingly, this variation is predominantly driven by a decrease in the long lifetime component $\tau_{2}$ , while the short lifetime component $\tau_{1}$ remains relatively unaltered with increasing pH. Placing these results in the context of cellular autofluorescence, there is a pool of NADH within the cytoplasm, where the pH is ∼7.2-7.4, and there is typically a larger pool within the mitochondria [52], where the pH is ∼8 [53]. The results from the two Datasets are in general consistent with one another when considering the measured experimental error for each Dataset, as can be seen visually from the size of the error bars shown in Fig. 2.

We have also observed an increase in NADH $\tau_{m}$ at pH 4 (Fig. S7), which to the best of our knowledge has not previously been reported. Previous studies have shown a decrease in NAD(P)H absorbance at low pH [54,55], which is the result of the degradation of the molecule in strong acid conditions. These studies are consistent with our observation of reduced fluorescence intensity and may explain the lifetime shift at pH 4. The time dependence of this transition that manifests in our data as an increasing fluorescence lifetime and decreasing intensity may indicate the solution’s equilibrium pushing further towards the non-fluorescent degradation product. However, the pHysiological relevance of these results is limited, as such low pH conditions are highly unusual for the intracellular environment.

In agreement with previous observations, the fluorescence signal of NADH generally increased in intensity and shifted towards shorter wavelengths with an increasing protein-bound fraction [14]. Increases in quantum yield and fluorescence lifetime in LDH-bound NADH have been attributed to the rigid orientation of NADH when bound [1] and on interaction between NADH and its binding site [56]. However, whereas the intensity of the fully-bound species remained high in Dataset 1 with increasing protein concentration, it decreased at high protein concentrations in Dataset 2 for LDH (Fig. 4(a)). This may be due to optical scattering in the solutions with the highest protein concentrations, but it remains unclear why this only affected Dataset 2. Further experimental repeats are required to investigate this effect in more detail. Regardless of the lower fluorescence intensity at the highest protein concentrations for Dataset 2, NADH $\tau_{m}$ and steady-state and time-resolved anisotropies increased with greater protein content as previously reported [27]. Differences in fluorescence lifetime at given protein-to-NADH ratios between the Datasets were not found to be greater than those differences between replicates of each individual Dataset. The large increase in rotational correlation time between the free species and the fully-bound species for LDH, which may be modelled as a monoexponential anisotropy decay, is in agreement with previously published analysis methodology and results [23].

We observed a consistently shorter lifetime at higher temperatures and note that the average lifetime difference between 25 and 37°C was larger for LDH-bound NADH (LDH ∼240 ps) compared to free NADH (∼85 ps, see Table S1 in Supplement 1). Our results suggest that for [LDH]:[NADH] of 0.25 (see Fig. 7(b)), NADH fluorescence lifetime increases slightly with pH. This is broadly consistent with a previous study reporting that the fluorescence lifetime of LDH-bound NADH is relatively insensitive to changes in pH over the range 6.1–10.8 [26]. The dissociation constant for the binding of LDH to NADH slightly increases with pH and slightly decreases with temperature over the ranges studied here [57]. An increase in dissociation constant would decrease the fraction of fluorescence from bound NADH giving a shorter mean fluorescence lifetime. Therefore, changes in dissociation constant with pH and temperature would be expected to affect the mean fluorescence lifetime in the opposite direction to the changes observed.

## 5. Conclusions

The main aim of this work was to report a systematic study of a range of microenvironmental parameters (pH, temperature and binding partner) on the fluorescence properties of NADH in aqueous solution, acquiring data using the same experimental system and fluorescence decay analysis approach for each dataset. In general, our work highlights the importance of controlling pH and temperature and provides an indication of the likely size of effect of any change in these parameters.

Our results are in broad agreement with previous studies and the key decay parameters for free NADH in aqueous solution are summarized in Table S1 in Supplement 1. At 25°C and 7.4 pH, the fluorescence decay parameters of free NADH were found to be 266 ± 25 ps, 781 ± 16 ps, 0.793 ± 0.023 and 482 ± 16 ps for *τ*_1_, *τ*_2_, *α*_1_ and the intensity weighted mean lifetime respectively. At 37°C and 7.4 pH, the equivalent values were found to be 252 ± 67 ps, 683 ± 221 ps, 0.81 ± 0.10 ps and 394 ± 54 ps, respectively. At 25°C, the average steady-state fluorescence anisotropy was 0.083 (average of Datasets 1 and 2) and the rotational correlation time was 409 ps (Dataset 2 only). When averaging the results of Dataset 1 and Dataset 2, we found the following rates of change: fluorescence intensity of NADH with temperature, -2.2%/^o^C; intensity-weighted mean fluorescence lifetime with temperature, -8 ps/^o^C; and steady-state anisotropy with temperature, -9.9 × 10^−4^/^o^C.

The fluorescence intensity of free NADH was reasonably constant above pH 6 at both 25°C and 37°C. Below pH 6, the fluorescence intensity decreased monotonically. The intensity-weighted mean fluorescence lifetime of free NADH was reasonably constant for pH 5 and above at both 25°C and 37°C. The intensity-weighted mean fluorescence lifetime increased at pH at 37°C.

For LDH-bound NADH, the fluorescence emission peak was blue-shifted by 19 nm compared to free NADH. At 37°C and pH 7.4, we observed a 2.5-fold increase in NADH fluorescence intensity when bound to LDH for [LDH]:[NADH] = 1. We believe that our fluorescence intensity measurements at higher protein concentrations were affected by optical scattering. Across both Datasets, the fluorescence decay parameters of fully LDH-bound NADH (i.e. [LDH]:[NADH] over the range 2-4) were found to be 804 ± 306 ps, 2503 ± 831 ps, 0.851 ± 0.068 and 1342 ± 237 ps for *τ*_1_, *τ*_2_, *α*_1_ and *τ_m_* respectively. At [LDH]:[NADH] = 4, the steady-state fluorescence anisotropy of LDH-bound NADH was found to be 0.32 and the rotational correlation time was 15,900 ± 2,100 ps.

We hope that this work may be able to aid the interpretation of *in vitro* and *in vivo* studies where NADH fluorescence plays a key role. The raw data from these experiments is freely available to download so that it can be analysed in other ways in the future. Our results show that it is important to control a range of experimental parameters in order to obtain consistent results, and we present results from two independent experiments to highlight the variability that can be expected using the approaches reported here.

This work could be extended to include other protein binding partners, as briefly reviewed in the introduction, that play relevant roles in glycolysis and oxidative metabolic pathways, as each one may affect NADH fluorescence differently. Furthermore, we believe it would be interesting to carry out a similar investigation to that presented here focusing on NADPH and also flavin adenine nucleotide (FAD) fluorescence, given its crucial role in metabolism together with NADH.

## Data Availability

The raw data from this work is available from Ref. [44].

## Supplemental document

See Supplement 1 for supporting content.
