# Supplementary material for: Characterization of NADH fluorescence properties under one-photon excitation with respect to temperature, pH, and binding to lactate dehydrogenase
Source: OSA Contin. 2021 May 10;4(5):1610–25. doi: 10.1364/OSAC.423082 (PMC8367293; doi:10.1364/OSAC.423082)
Supplement: Supplementary file 1 [file osac-4-5-1610-s001.pdf]

Supplemental Document

OSA CONTINUUM

Characterization of NADH fluorescence properties under one-photon excitation with respect to temperature, pH, and binding to lactate dehydrogenase: supplement

TAYLOR M. CANNON,[1,4] JOAO L. LAGARTO,[1,4] BENJAMIN T. DYER,[2] EDWIN GARCIA,[1] DOUGLAS J. KELLY,[1] NICHOLAS S. PETERS,[2] ALEXANDER R. LYON,[2] PAUL M. W. FRENCH,[1] AND CHRIS DUNSBY[1,3,*]

[1] *Department of Physics, Imperial College London, London, SW7 2AZ, UK*
[2] *National Heart and Lung Institute, Imperial College London, Du Cane Road, London, W12 0NN, UK*
[3] *Centre for Pathology, Imperial College London, London, W12 0NN, UK*
[4] *These authors contributed equally to this work and are listed in alphabetical order*
[*] *christopher.dunsby@imperial.ac.uk*

This supplement published with The Optical Society on 10 May 2021 by The Authors under the terms of the Creative Commons Attribution 4.0 License in the format provided by the authors and unedited. Further distribution of this work must maintain attribution to the author(s) and the published article's title, journal citation, and DOI.

Supplement DOI: https://doi.org/10.6084/m9.figshare.14473092

Parent Article DOI: https://doi.org/10.1364/OSAC.423082

Characterisation of NADH fluorescence properties under one-photon excitation with respect to temperature, pH and binding to lactate dehydrogenase: supplemental document

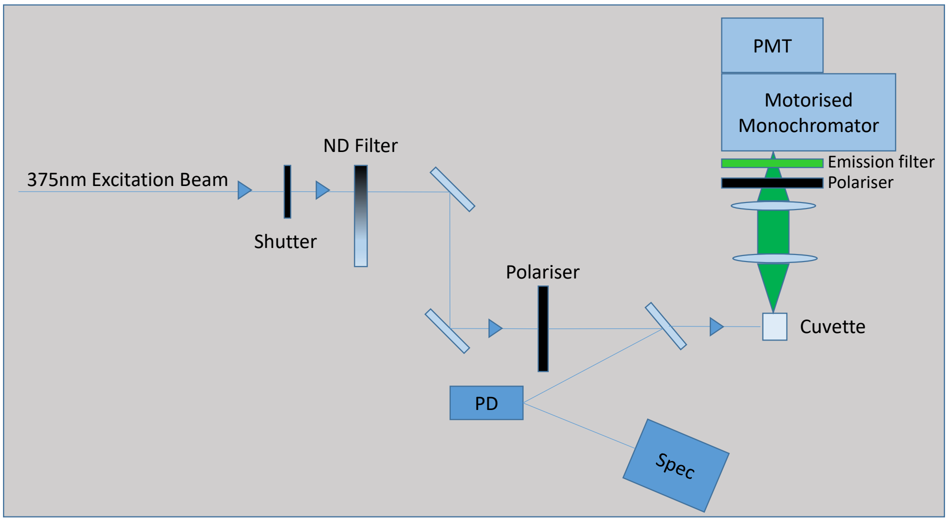

Fig. S1. Diagram of the cuvette-based time-resolved spectroscopy system. ND – neutral density, PD – photodiode, Spec – spectrometer, PMT – photomultiplier tube.

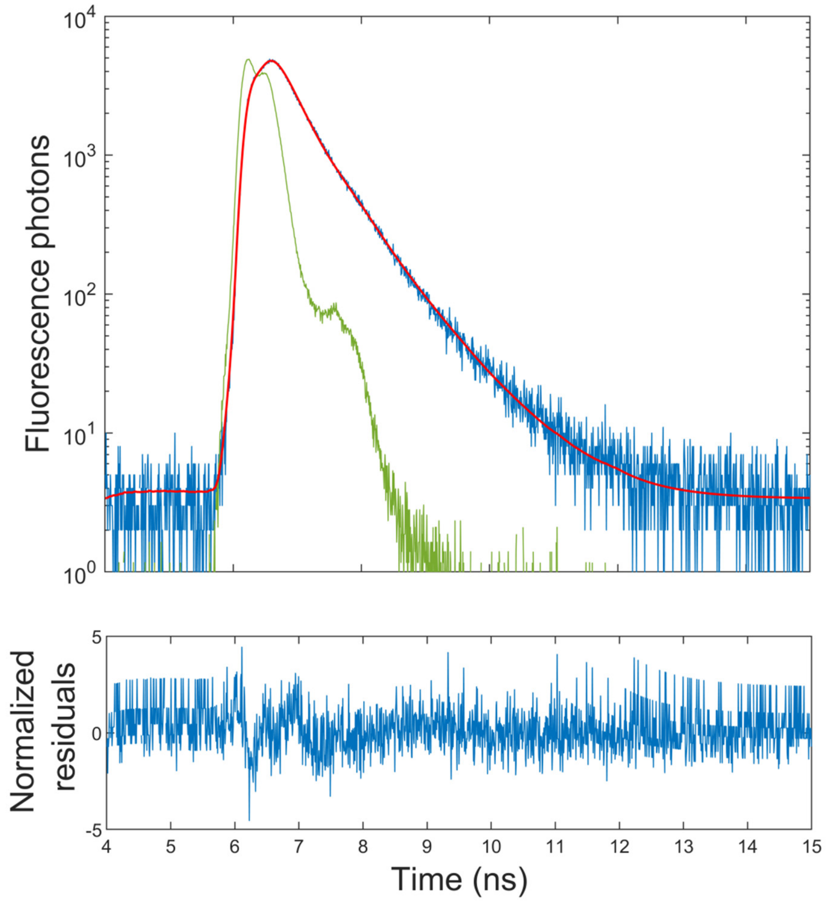

Fig. S2. Exemplar fluorescence decay from Dataset 1 and fit to data for free NADH at pH 7.4 at 25°C. Top panel: raw data (blue), fit to data (red) and IRF (green). Bottom panel: normalised residuals.

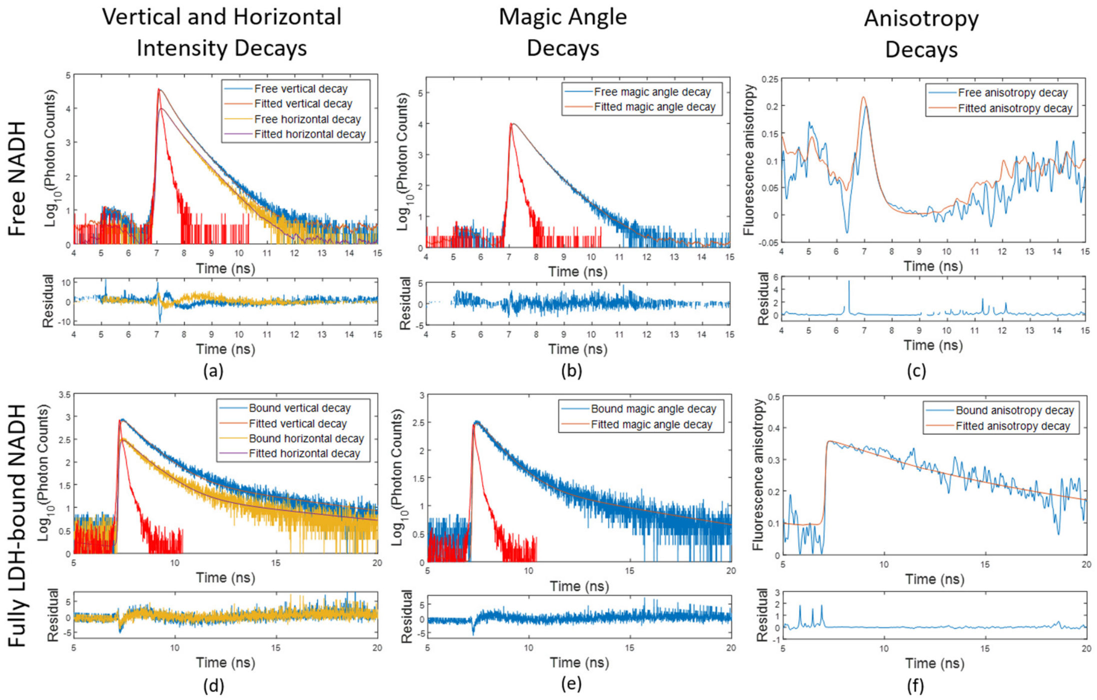

Fig. S3. Free and LDH-bound NADH time-resolved anisotropy analysis. Vertical, horizontal, calculated magic angle, and anisotropy decays are shown for free NADH (a-c) and LDH-bound NADH ([LDH]:[NADH] = 4) (d-f), along with their respective fitted curves. The FLIM*fit* software was used to globally fit the parallel and perpendicular decays for each sample (left hand column) simultaneously. The middle and right-hand columns show the magic angle and anisotropy decays and fits calculated by FLIMfit. All intensity decay curves are plotted on a logarithmic scale.

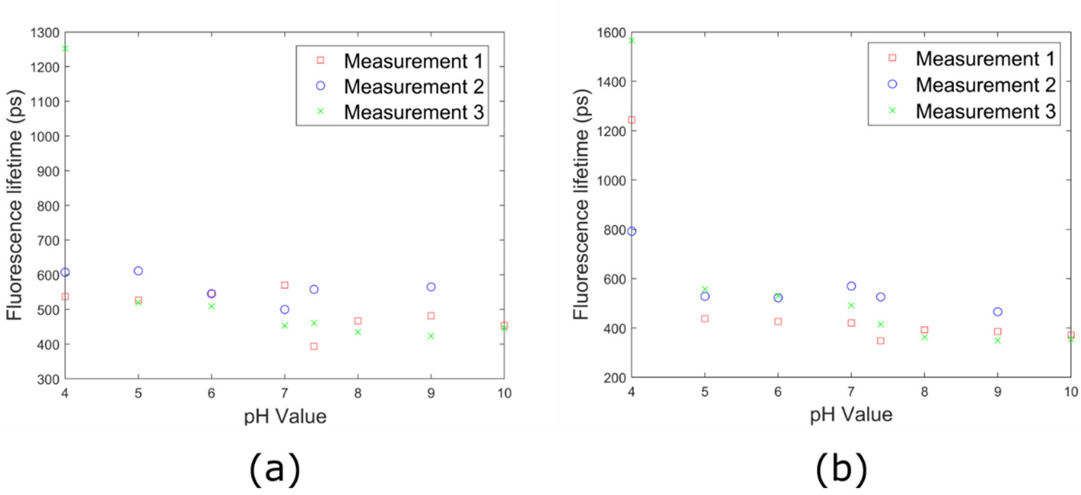

Fig. S4. Comparison of experimental measurements in Dataset 2 under varying pH at a) 25ºC and b) 37ºC. Although some variation occurred between measurements performed under the same conditions on different days, the overall responses to the changing environment showed the same trends for each parameter studied. The HCl and NaOH titration pH-adjustment method was used for Measurements 1 and 2 and the Chemvelope buffer method was used for Measurement 3.

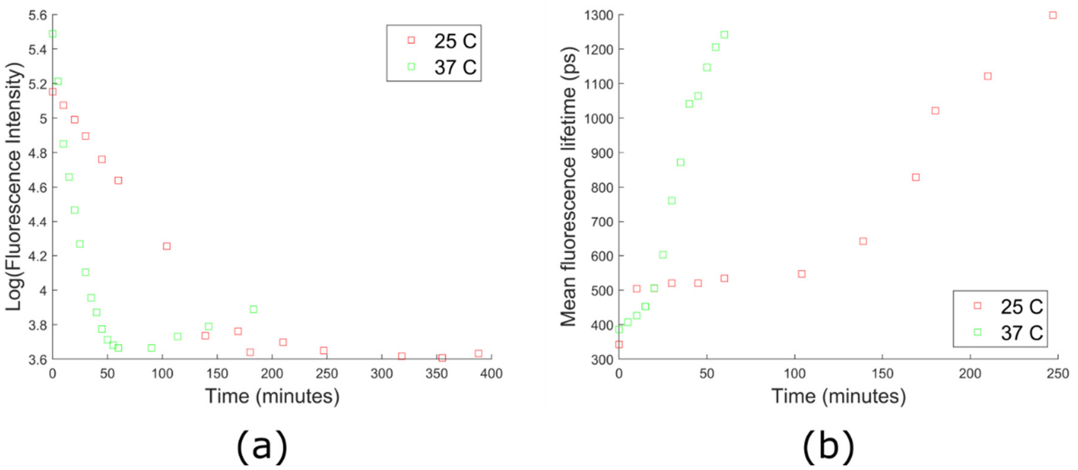

Fig. S5. Change in NADH fluorescence properties over time at pH 4. (a) Fluorescence intensity and (b) fluorescence lifetime as a function of time at 25°C and 37°C.

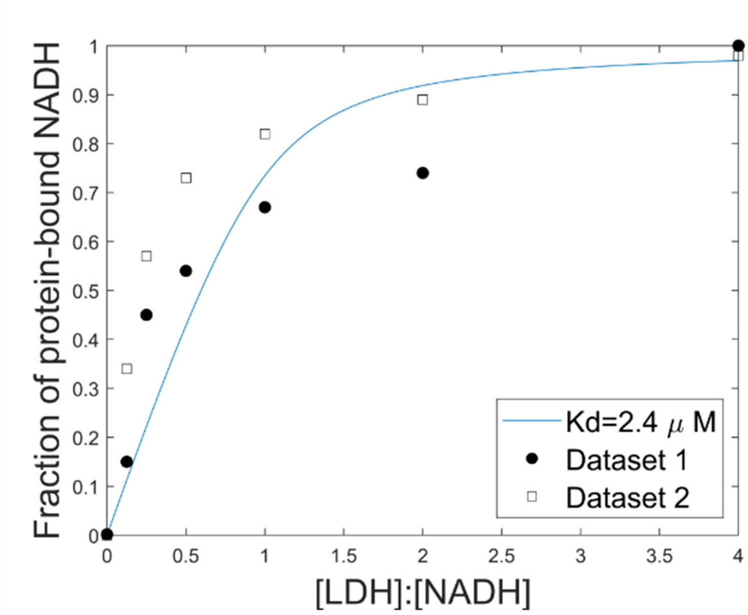

Fig. S6. Comparison between theoretical protein bound fraction models (lines) and measured data (discrete data points). The expected fraction of protein-bound NADH was calculated for the association of LDH with NADH at varying protein concentrations. The calculation accounted for the multiple binding sites for LDH to which NADH can bind and used dissociation constants found in literature [1]. The concentration of NADH was held constant at 12.5 μM.

Table S1. Summary of NADH fluorescence lifetime measurements under varying conditions

Conditions at 25°C	τ_1 (ps)	τ_2 (ps)	α_1	Mean lifetime (ps)	Number of measurements
7.4 pH, no protein, temperature experiments	248±161	792±395	0.777± 0.091	493±125	4
7.4 pH, no protein, pH experiments	284±86	770±165	0.809± 0.034	471±83	3
Mean± standard deviation over independent experiments †	**266±25**	**781±16**	**0.793± 0.023**	**482±16**	**2**
Conditions at 37°C					
7.4 pH, no protein, temperature experiments	257±57	610±120	0.800± 0.099	377±23	4
7.4 pH, no protein, pH experiments	248±54	694±193	0.811± 0.051	415±79	4
7.4 pH, no protein, LDH experiments	252±90	747±350	0.827± 0.157	390 ±61	2
Mean± standard deviation over independent experiments †	**252±5**	**684±69**	**0.81± 0.01**	**394±19**	**3**

† In the rows shown in normal font, the standard deviation is calculated over repeat measurements for that condition. In the rows shown in bold font, the standard deviation is calculated over the different groups of measurements for that temperature, which are shown in the rows above.

Table S2. Emission spectra peak wavelengths at varying protein to NADH ratios

	**LDH (nm)**
[Protein]:[NADH]=4:1	442
[Protein]:[NADH]=1:4	454
Free NADH	461

References

1. Fromm, H.J. *Determination of Dissociation Constants of Coenzymes and Abortive Ternary Complexes with Rabbit Muscle Lactate Dehydrogenase from Fluorescence Measurements*. J Biol Chem, 1963. **238**(9): p. 2938-2944.
